# Investigation of Possible Intraoperative Transmission of *Brucella melitensis*, Slovenia

**DOI:** 10.3201/eid3110.250587

**Published:** 2025-10

**Authors:** Igor Potparić, Klemen Bošnjak, Jana Avberšek, Bojan Papić, Petra Bogovič, Polona Maver Vodičar, Martin Sagadin, Mateja Pirš, Miša Korva, Tatjana Avšič-Županc, Miha Vodičar

**Affiliations:** University Medical Centre Ljubljana, Ljubljana, Slovenia (I. Potparić, K. Bosnjak, P. Bogovič, M. Vodicar); University of Ljubljana, Ljubljana (K. Bošnjak, J. Avberšek, B. Papić, P. Maver Vodičar, M. Sagadin, M. Pirš, M. Korva, T. Avšič-Županc, M. Vodičar)

**Keywords:** *Brucella melitensis*, intraoperative transmission, whole-genome sequencing, spondylodiscitis, spondylitis, zoonoses, bacteria, aerosol transmission, Slovenia

## Abstract

We report possible intraoperative transmission of *Brucella melitensis* in Slovenia, likely caused by aerosolized particles during wound irrigation. Whole-genome multilocus sequence typing revealed that isolates from the patient and the surgeon belonged to the same transmission cluster, differing by 1 allele. Our findings raise awareness of occupational risks faced by orthopedic surgeons.

Brucellosis causes >500,000 reported cases worldwide and is endemic in the Balkans, Central Asia, India, Central and South America, and the Middle East (*1*,*2*). Brucellosis is caused by gram-negative intracellular bacteria of the genus *Brucella*, most often *Brucella melitensis* (*1*). The primary routes of infection include having direct contact with infected animals, consuming unpasteurized dairy products or or undercooked meat of infected animals, and, in rare cases, aerosol inhalation (*3*,*4*). 

The prevailing symptoms of brucellosis are fever, night sweats, arthralgia, malaise, headache, and fatigue. Because of nonspecific symptoms and often prolonged incubation period, brucellosis presents a diagnostic challenge, particularly for healthcare providers in nonendemic areas where cases occur sporadically (*1*–*3*). Infection frequently affects osteoarticular structures; spinal involvement is estimated in 54% of brucellosis cases, mainly manifesting as spondylitis or spondylodiscitis (*3*,*5*). Animal-to-human transmission is most frequent in veterinary and agricultural workers; however, direct patient-to-patient transmission is rare. Laboratory workers face an increased occupational risk; most reported cases are linked to handling *Brucella* isolates on an open laboratory bench (*6*). 

In Slovenia, brucellosis cases are rare and mostly imported from the Balkans. Healthcare workers reported only 12 cases of brucellosis in Slovenia during 2017−2022, with 6 of those cases diagnosed in 2019 (*7*). We report possible intraoperative transmission of *B. melitensis* from patient to surgeon in Slovenia.

## The Study

A 64-year-old woman with a history of back pain sought treatment at University Medical Center Ljubljana (Ljubljana, Slovenia), where physicians observed acute-onset left-sided femoralgia and motor deficit in the left foot dorsiflexion. Magnetic resonance imaging revealed a herniated disk at L4–5 and L5 neuroforaminal stenosis, prompting left-sided L4–5 discectomy and L5 foraminotomy. Postoperatively, the patient reported only a slight reduction in pain, despite uncomplicated rehabilitation. At 1-year follow-up, the patient reported persistent pain, following the preoperative pattern. Follow-up imaging, including radiography and magnetic resonance imaging, revealed a collapsed L4–5 disk and L4 anterolisthesis, accompanied by a minor seroma at the surgical site. Thus, surgeons performed an L4–5 posterior spinal fusion 20 months after the initial surgery, yielding a satisfactory recovery and hospital discharge of the patient 1 week after surgery. 

At 1-month follow-up, physicians observed persistent serous wound drainage, and laboratory results revealed slightly elevated levels of C-reactive protein (20 mg/L; reference range <10 mg/L) and erythrocyte sedimentation rate (58 mm/h; reference range <30 mm/h). Leukocyte count remained unremarkable (7.7 × 10^9^ cells/L; reference range 4.5–11.0 × 10^9^ cells/L). Surgeons performed a revision surgery with microbiological sampling, administering amoxicillin with clavulanic acid and ciprofloxacin empirically. Laboratory analysis of a subcutaneous tissue sample identified *B. melitensis*, which was further confirmed by real-time PCR (Appendix). Results of blood analysis showed *Brucella* IgM and IgG. On the basis of those results, treating physicians changed the antibiotic regimen to intravenous gentamicin (240 mg/d) and oral doxycycline (200 mg/d) for 14 days, followed by doxycycline (200 mg/d) and rifampin (900 mg/d) for 6 months. C-reactive protein levels returned to reference range after 1 month. 

**Figure 1:**
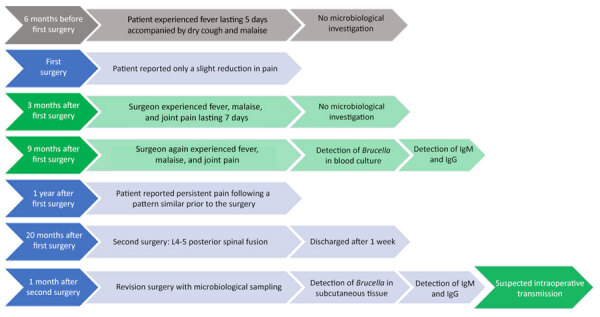
Patient-to-surgeon transmission timeline in study investigating possible intraoperative transmission of *Brucella melitensis,* Slovenia.

Detailed retrospective analysis of the patient’s medical history revealed that she frequently traveled to rural areas of Bosnia and Herzegovina and occasionally consumed unpasteurized cow milk and homemade goat cheese. Six months before the initial surgery, during one of those visits, she had a 5-day fever accompanied by dry cough and malaise (Figure 1).

Three months after the patient’s initial surgery, the lead surgeon experienced fever, malaise, and joint pain lasting 7 days, initially attributed to a viral infection. He underwent no microbiologic investigation. Nine months later, the surgeon experienced a recurrence of fever, malaise, and joint pain; symptoms persisted and gradually worsened over 9 days. Given his recent travel to Moldova, the surgeon underwent a comprehensive microbiologic investigation, revealing *B. melitensis* in blood cultures and *Brucella* spp. antibodies in the blood sample. After receiving the established antibiotic regimen, he recovered without complications. Of note, the surgeon had no travel history to endemic regions in the previous 2 years, except for Moldova, and recalled no food source of infection. Retrospective serologic investigation revealed the absence of *Brucella* spp. antibodies in a blood sample taken 1 month before the initial surgery. Thus, when the surgeon’s laboratory results showed *B. melitensis* infection following the third revision surgery, retrospective analysis indicated that transmission might have occurred during the first operation. Surgical staff involved in any of the operations underwent serologic testing, revealing IgG in 1 additional asymptomatic person, who subsequently received antibiotic treatment.

**Figure 2:**
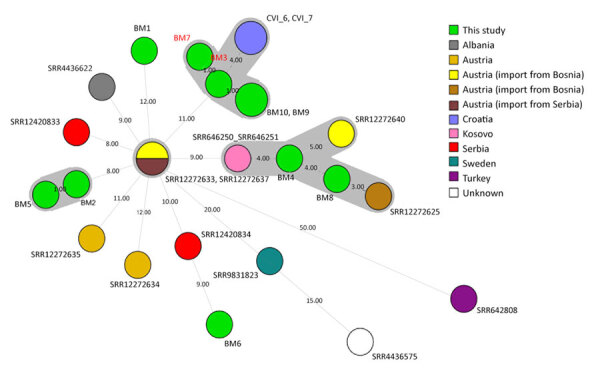
Minimum spanning tree based on whole-genome multilocus sequence typing allele profiles showing the relatedness of 25 *Brucella melitensis* isolates in study investigating possible intraoperative transmission of *Brucella melitensis*, Slovenia. Numbers above the branches correspond to the number of allele differences. Gray shading indicates clusters of isolates differing by ≤6 alleles. Isolates sequenced in this study shown in green: BM1 (National Center for Biotechnolgoy Information Sequence Read Archive accession no. SRR32696437), BM2 (accession no. SRR32696436), BM4 (accession no. SRR32696434), BM5 (accession no. SRR32696433), BM7 (red text, the patient; accession no. SRR32696431), BM9 (accession no. SRR32696429), BM10 (imported from Bosnia and Herzegovina; accession no. SRR32696428); BM8 (imported from Croatia; accession no. SRR32696430), BM6 (imported from North Macedonia; accession no. SRR32696432), and BM3 (red text, the surgeon; accession no. SRR32696435).

To confirm this suspected patient-to-surgeon transmission of *B. melitensis*, we performed whole-genome sequencing (WGS) on the isolates from both the patient and the surgeon, along with 8 additional human *B. melitensis* isolates identified in Slovenia during 2017−2020. To determine the putative geographic origin of the Slovenia isolates, we included in the analysis 15 closely related (differing by <80 alleles from the Slovenia isolates), publicly available *B. melitensis* sequence type 8 genomes belonging to the Eastern Mediterranean clade (genotype II) (Appendix Table) (*8*). All Slovenia isolates were sequence type 8. We observed 3 whole-genome multilocus sequence typing (wgMLST) clusters (Figure 2), of which 2 also encompassed publicly available but epidemiologically unrelated isolates from neighboring countries. Two isolates formed no clusters. The isolates from the patient (BM7) and the surgeon (BM3) formed a common cluster, differing by only 1 wgMLST allele (Figure 2). That cluster also encompassed 2 other isolates (BM9 and BM10) from Bosnia and Herzegovina and was most closely related (≥4 wgMLST allele differences) to the isolates from Croatia (CVI_6 and CVI_7). The patients from isolates BM7, BM9, and BM10 reported travel history to Bosnia and Herzegovina, so we cannot exclude the possibility that their infections are related. However, the surgeon (isolate BM3) neither traveled to the Western Balkans (including Bosnia and Herzegovina) nor consumed milk or cheese made from unpasteurized milk from that region.

## Conclusions

Most documented cases of brucellosis result from high-risk exposures or laboratory-acquired infections (*1*,*6*,*7*); knowledge is limited regarding exposure to *Brucella* spp. in other hospital settings (*8*,*9*). Although the surgeon in this report strictly followed standard intraoperative infection control measures, we suspect patient-to-surgeon transmission occurred during a discectomy, when wound irrigation caused small droplets to aerosolize, producing an environment in which a standard surgical mask provided insufficient protection. 

The overall incidence of human brucellosis in Europe has declined substantially over the past decade, but the Mediterranean region remains highly affected (*7*) (Appendix). Furthermore, brucellosis is difficult to diagnose early because of nonspecific symptoms (*10*), and the infected tissue is nonpyogenic, meaning the infection can easily be overlooked in the absence of epidemiologic suspicion. In this case, it was only after the third operation, after brucellosis in the patient had been confirmed, that a retrospective review of magnetic resonance imaging suggested subtle signs of a possible low-grade infection. In similar cases, it would be prudent to assess the patient’s serostatus and initiate appropriate antibiotic treatment before surgery and to ensure surgical staff uses appropriate personal protective equipment to reduce the risk of aerosol transmission.

Although WGS has discriminatory power to trace outbreaks and investigate human-to-human transmission (*11*,*12*), this type of analysis can be limited by small numbers of *Brucella* isolates available for WGS typing. The scarcity of isolates in this study primarily resulted from low incidence of confirmed cases of human brucellosis in Slovenia, as well as the absence of isolates from other sources (e.g., food) collected within the same geographic region and time period. Nevertheless, our study highlights the effectiveness of WGS in confirming that, although all Slovenia isolates were closely related *B. melitensis* genotype II isolates, only the isolates from the patient and surgeon were almost identical. This specific finding underscores the potential risk of *B. melitensis* transmission in the context of orthopedic surgery.

Appendix Additional information for investigation of possible intraoperative transmission of *Brucella melitensis*, Slovenia.
