# Supplementary material for: Investigation of Possible Intraoperative Transmission of Brucella melitensis, Slovenia
Source: Emerg Infect Dis. 2025 Oct;31(10):2034–7. doi: 10.3201/eid3110.250587 (PMC12483101; doi:10.3201/eid3110.250587)
Supplement: Appendix — Additional information for investigation of possible intraoperative transmission of Brucella melitensis, Slovenia. [file 25-0587-Techapp-s1.pdf]

Article DOI: http://doi.org/10.3201/eid3110.250587

EID cannot ensure accessibility for supplementary materials supplied by authors. Readers who have difficulty accessing supplementary content should contact the authors for assistance.

Investigation of Possible Intraoperative Transmission of *Brucella melitensis*, Slovenia

Appendix

Methods

Nucleic acid extraction

For routine molecular diagnostics, nucleic acid was extracted from positive blood culture or homogenized tissue sample. In both cases, 200 µL of clinical sample was mixed with 200 µL of ATL buffer (Qiagen, Hilden, Germany), followed by nucleic acid extraction using the EZ1&2 Virus Mini Kit v2.0 (Qiagen, Hilden, Germany) according to the manufacturer's instructions.

Real-time PCR

Brucella melitensis DNA was detected by modified real-time PCR by previously described methods (*1*). Real-time RT-PCR was performed on a QuantStudio Pro 7 system (Applied Biosystems, Thermo Fisher Scientific, USA). Reactions were carried out in a total volume of 20 µL, containing 5 µL of DNA, 5 µL of TaqMan Fast Virus 1-Step Master Mix (Applied Biosystems, Thermo Fisher Scientific, Grand Island, NY, USA), 1 µM of each primer (Bruc F 5′-gCTCgACACAAAgggCCA-3′ and Bruc R 5′-CAAgCgTggTCTggCgA-3′), 0.2 µM of probe (Bruc-P 5′-FAM-CCGAGATACAAA-MGB-BHQ1–3′) and water. Cycling conditions were as follows: 95°C for 20 s, followed by 40 cycles of 95°C for 3 s and 60°C for 30 s.

Serology

The presence of specific IgG and IgM antibodies against *Brucella* spp. was detected using an indirect immunofluorescence assay (*Brucella* MIF IgG and IgM kits; Fuller Laboratories, Fullerton, CA, USA) and enzyme-linked immunosorbent assay (Euroimmun, Lubeck, Germany). Both tests were performed according to the manufacturer's instructions.

Bacterial cultivation

Brucella cultivation was performed in a biosafety level 3 laboratory. Clinical samples (seven positive blood cultures, one knee joint puncture, one shoulder joint puncture and one homogenized subcutaneous tissue) were inoculated on blood agar (BAB) and trypticase soy agar (TSA) plates and incubated for 4 days at 37°C. Thereafter, small, light-gray colonies were observed and *B. melitensis* was confirmed by real-time PCR.

DNA extraction from bacterial isolates

For whole-genome sequencing (WGS), presumptive *Brucella* spp. colonies were suspended in 200 μL of phosphate buffered saline (PBS), and DNA was extracted using the High Pure PCR Template Preparation Kit (Roche Diagnostics, Mannheim, Germany) according to the manufacturer's instructions for Isolation of nucleic acids from bacteria.

WGS and bioinformatics analysis

Sequencing was performed on an Illumina MiSeq (2 × 300 bp) or NovaSeq 6000 (2 × 150 bp) platform according to the manufacturer's instructions. Raw reads were imported into BioNumerics software v7.3.1 (Applied Maths NV, Belgium). To ensure high-quality data, only reads and assemblies that passed the quality parameters used in the wgMLST quality assessment window were included in further analysis. Assembly-based and assembly-free allele calls were performed, and the wgMLST minimum spanning tree was constructed by applying the *B. melitensis*-specific subscheme consisting of 4,217 loci, with allele calls considered as categorical data. Based on the observed genetic distances and the origin of the isolates under study, a previously proposed threshold of ≤6 allele differences was considered appropriate and was used for cluster delineation (*2*). *In silico* MLST was performed in BioNumerics by applying the 9- and 21-loci *Brucella* MLST schemes, which are synchronised with nomenclature implemented in *Brucella* PubMLST Web site (https://pubmlst.org/brucella/) (*3*).

Appendix Table. Isolate metadata. Ten human clinical *B. melitensis* isolates collected from 2017–2020 were included in the study.

Key	*Isolate*	*Year*	*PubMLST 9/21 loci ST*	*Source*	*NCBI SRA*	*Origin*	*Reference*
BM1	1/17	2017	ST8/ST8	Human	SRR32696437	Slovenia (ex. BiH)	This study
BM2	2/17	2017	ST8/ST8	Human	SRR32696436	Slovenia (ex. BiH)	This study
BM3	1/18	2018	ST8/ST8	Human	SRR32696435	Slovenia	This study
BM4	1/19	2019	ST8/ST8	Human	SRR32696434	Slovenia (ex. BiH)	This study
BM5	2/19	2019	ST8/ST8	Human	SRR32696433	Slovenia (ex. BiH)	This study
BM6	3/19	2019	ST8/ST8	Human	SRR32696432	Slovenia (ex. North Macedonia)	This study
BM7	4/19	2019	ST8/ST8	Human	SRR32696431	Slovenia (ex. BiH)	This study
BM8	5/19	2019	ST8/ST8	Human	SRR32696430	Slovenia (ex. Croatia)	This study
BM9	1/20	2020	ST8/ST8	Human	SRR32696429	Slovenia (ex. BiH)	This study
BM10	2/20	2020	ST8/ST8	Human	SRR32696428	Slovenia (ex. BiH)	This study
SRR4436622	BwIM_ALB_46	2015	ST8/ST8	Human	CP018480.1, CP018481.1; SRR4436622	Albania	(*4*)
SRR4436575	BwIM_XXX_12	2015	ST8/ST8	Human	SRR4436575	Unknown	(*4*)
SRR646250/51	F8/01–155	2001	ST8/ST8	Cattle	SRR646250, SRR646251	Kosovo	(*4*)
SRR642808	F9/05	2005	ST8/ST8	Human	SRR642808	Turkey	(*5*)
SRR9831823	2017-TE-24378–1-13	2000	ST8/ST8	Human	SRR9831823	Sweden	(*6*)
SRR12272625	511504	2016	ST8/ST8	Human	SRR12272625	Austria (ex. Croatia)	(*7*)
SRR12272633	511512	2012	ST8/NA	Human	SRR12272633	Austria (ex. Serbia)	(*7*)
SRR12272634	511511	2005	ST8/ST8	Human	SRR12272634	Austria	(*7*)
SRR12272635	511510	2011	ST8/ST8	Human	SRR12272635	Austria	(*7*)
SRR12272637	511508	2011	ST8/ST8	Human	SRR12272637	Austria (ex. BiH)	(*7*)
SRR12272640	511496	2017	ST8/ST8	Human	SRR12272640	Austria (ex. BiH)	(*7*)
SRR12420833	510333	Unknown	ST8/ST8	Small ruminant	SRR12420833	Serbia	(*7*)
SRR12420834	510332	Unknown	ST8/ST8	Small ruminant	SRR12420834	Serbia	(*7*)
CVI_6		2018	ST8/ST8	Cattle	CP058599.1, CP058600.1	Croatia	(*8*)
CVI_7		2018	ST8/ST8	Cattle	CP058597.1, CP058598.1	Croatia	(*8*)

References

1. Kaden R, Ferrari S, Alm E, Wahab T. A novel real-time PCR assay for specific detection of *Brucella melitensis*. BMC Infect Dis. 2017;17:230. PubMed https://doi.org/10.1186/s12879-017-2327-7

2. Janowicz A, De Massis F, Ancora M, Cammà C, Patavino C, Battisti A, et al. Core genome multilocus sequence typing and single nucleotide polymorphism analysis in the epidemiology of *Brucella melitensis* infections. J Clin Microbiol. 2018;56:e00517–18. PubMed https://doi.org/10.1128/JCM.00517-18

3. Jolley KA, Bray JE, Maiden MCJ. Open-access bacterial population genomics: BIGSdb software, the PubMLST.org website and their applications. Wellcome Open Res. 2018;3:124. PubMed https://doi.org/10.12688/wellcomeopenres.14826.1

4. Georgi E, Walter MC, Pfalzgraf MT, Northoff BH, Holdt LM, Scholz HC, et al. Whole genome sequencing of *Brucella melitensis* isolated from 57 patients in Germany reveals high diversity in strains from Middle East. PLoS One. 2017;12:e0175425. PubMed https://doi.org/10.1371/journal.pone.0175425

5. Pisarenko SV, Kovalev DA, Volynkina AS, Ponomarenko DG, Rusanova DV, Zharinova NV, et al. Global evolution and phylogeography of *Brucella melitensis* strains. BMC Genomics. 2018;19:353. PubMed https://doi.org/10.1186/s12864-018-4762-2

6. Sacchini L, Wahab T, Di Giannatale E, Zilli K, Abass A, Garofolo G, et al. Whole genome sequencing for tracing geographical origin of imported cases of human brucellosis in Sweden. Microorganisms. 2019;7:e398. PubMed https://doi.org/10.3390/microorganisms7100398

7. Schaeffer J, Revilla-Fernández S, Hofer E, Posch R, Stoeger A, Leth C, et al. Tracking the origin of Austrian human brucellosis cases using whole genome sequencing. Front Med (Lausanne). 2021;8:635547. PubMed https://doi.org/10.3389/fmed.2021.635547

8. Špičić S, Zdelar-Tuk M, Ponsart C, Hendriksen RS, Reil I, Girault G, et al. New *Brucella* variant isolated from Croatian cattle. BMC Vet Res. 2021;17:126. PubMed https://doi.org/10.1186/s12917-021-02833-w
